# Identifying frailty: do the Frailty Index and Groningen Frailty Indicator cover different clinical perspectives? a cross-sectional study

**DOI:** 10.1186/1471-2296-14-64

**Published:** 2013-05-21

**Authors:** Irene Drubbel, Nienke Bleijenberg, Guido Kranenburg, René JC Eijkemans, Marieke J Schuurmans, Niek J de Wit, Mattijs E Numans

**Affiliations:** 1 Department of General Practice, Julius Center for Health Sciences and Primary Care, University Medical Center Utrecht, Universiteitsweg 100, Utrecht 3584 CG, The Netherlands; 2 Department of Biostatistics, Julius Center for Health Sciences and Primary Care, University Medical Center Utrecht, Universiteitsweg 100, Utrecht, 3584 CG, The Netherlands; 3 Department of Rehabilitation, Nursing Science and Sports, University Medical Center Utrecht, Heidelberglaan 100, Utrecht, 3584 CX, The Netherlands

**Keywords:** Frailty, Primary care, Frailty index, Groningen frailty indicator, Proactive care

## Abstract

**Background:**

Early identification of frailty is important for proactive primary care. Currently, however, there is no consensus on which measure to use. Therefore, we examined whether a Frailty Index (FI), based on ICPC-coded primary care data, and the Groningen Frailty Indicator (GFI) questionnaire identify the same older people as frail.

**Methods:**

We conducted a cross-sectional, observational study of 1,580 patients aged ≥ 60 years in a Dutch primary care center. Patients received a GFI questionnaire and were surveyed on their baseline characteristics. Frailty-screening software calculated their FI score. The GFI and FI scores were compared as continuous and dichotomised measures.

**Results:**

FI data were available for 1549 patients (98%). 663 patients (42%) returned their GFI questionnaire. Complete GFI and FI scores were available for 638 patients (40.4%), mean age 73.4 years, 52.8% female. There was a positive correlation between the GFI and the FI (Pearson’s correlation coefficient 0.544). Using dichotomised scores, 84.3% of patients with a low FI score also had a low GFI score. In patients with a high FI score, 55.1% also had a high GFI score. A continuous FI score accurately predicted a dichotomised GFI score (AUC 0.78, 95% CI 0.74 to 0.82). Being widowed or divorced was an independent predictor of both a high GFI score in patients with a low FI score, and a high FI score in patients with a low GFI score.

**Conclusions:**

The FI and the GFI moderately overlap in identifying frailty in community-dwelling older patients. To provide optimal proactive primary care, we suggest an initial FI screening in routine healthcare data, followed by a GFI questionnaire for patients with a high FI score or otherwise at high risk as the preferred two-step frailty screening process in primary care.

## Background

The frail, older population introduces a heavy burden on primary health care [1-3]. To improve proactive care for this vulnerable group, various frailty measures have been suggested. However, there is a lack of consensus on which measure to use in routine primary care practice [4-7].

One way of assessing frailty in the primary care setting is with a Frailty Index (FI), which uses readily available data [8]. When interfaced with a patient information database, FI software will automatically screen patients for so-called ‘health deficits’, including symptoms, diseases, or impairments. The proportion of identified deficits to those in the predefined list is the resulting FI score, a dynamic state variable that adequately reflects the frailty level of an individual [9,10]. Alternatively, another approach to measure frailty in the primary care setting is with a self-assessment questionnaire, such as the 15-item Groningen Frailty Indicator (GFI). The GFI questionnaire screens for self-reported limitations and is widely used in The Netherlands [11]. Higher scores indicate higher frailty levels and an increased need for integrated care [12].

Both the FI and the GFI are feasible for use in primary care. To compute an FI score, center-specific software is needed, which requires financial investment for development and training. Thereafter, limited time is necessary for the generation of frailty reports from the Electronic Medical Records (EMR) data. Conversely, implementation of the GFI questionnaire requires less start-up expenses, but the post-screening process is more time demanding. Apart from logistical differences, also the clinical perspective of these two measures may be different. Whereas the FI score predicts patients’ risk of adverse health outcomes, the GFI score reflects current problems in patients’ daily lives. To our knowledge, no previous study has examined whether these frailty measures, regardless of individual focus, will identify the same population as frail [13]. Therefore, the aim of this study is to assess if, in community-dwelling older adults, an FI based on ICPC- and ATC-coded routine primary care data and the GFI will identify the same older patients as frail [14,15].

## Methods

### Ethical approval

This study was approved by the Institutional Review Board of the University Medical Center Utrecht, The Netherlands (reference number 10-149/O). Written informed consent was obtained from all patients.

### Design

Cross-sectional, observational study conducted in a primary care setting.

### Setting

Patients were enrolled from an urban primary care center with seven general practitioners (GPs) managing 10,500 patients in Utrecht, The Netherlands.

### Participants

Participants were selected from the center’s electronic medical record (EMR) data file. The EMR contained patient information dated through 20 May 2011. All patients 60 years of age and older were eligible for inclusion in the study.

### Procedures

On 9 May 2011, the GPs sent all eligible patients a patient information letter, an informed consent form, and a questionnaire. This questionnaire consisted of the GFI (see Additional file 1: GFI) as well as questions regarding age, sex, ethnicity, education, and marital status. Patients were included if they returned the informed consent form and questionnaire within three weeks. No reminders were sent.

Concurrent to the mailing of questionnaires, frailty-screening software was interfaced with an anonymous EMR data file to calculate the FI score for each patient. Additionally, this software systematically extracted data on age, gender, and consultation gap, defined as the total number of days from a patient’s last contact with the GP until the EMR snapshot date. This timeframe was determined by searching for the most recently registered ICPC code, with the exception of influenza vaccination. We only considered the gap till the last consultation, and did not include earlier consultation patterns. In general, age, gender, and care avoidance are related to frailty and a greater risk of adverse health outcomes [16,17]. Therefore, in addition to the aforementioned questions, these parameters were included as baseline characteristics for our population.

The frailty-screening software uploaded the EMR data to a highly protected server where frailty reports were created prior to being routed to the primary care center. During this process, an external ‘trusted third party’ routing created pseudonyms to encode personal data so that data processing was completely anonymous outside the primary care center. Included patients consented to the procedure that the researchers would ask the primary care center for all variables that the frailty-screening software calculated. Frailty report data for the remaining patients of the primary care center were anonymously released into a non-responder data file.

### Measurements

#### GFI

The GFI is a validated, 15-item questionnaire with a score range from zero to fifteen that assesses the physical, cognitive, social, and psychological domains. A GFI score of four or greater is considered the cut-off point for frailty [11]. The GFI has demonstrated high internal consistency and construct validity when compared to the Tilburg Frailty Indicator and the Sherbrook Postal Questionnaire [13].

#### Frailty index

We used an FI that we developed in a previous FI validation study in the same primary care center [18]. In short, we first selected 140 relevant ICPC-coded items and an ATC-coded polypharmacy item. This selection was based on the literature on FI construction, data on age-related deficit prevalence and health burdens, and a consensus meeting with a local expert group of GPs [19-22]. The ICPC-coded items reflect a range of symptoms, diseases, functional impairments and social problems. Second, to reach a deficit prevalence of at least 5%, we arranged these items into single- and multi-item deficits (see Additional file 2: FI deficits). Being aware of the commonly employed lower limit for deficit prevalences of 1%, we opted for 5% because of the relatively low prevalence of our separate ICPC-coded items. Furthermore, multi-item deficits needed to reflect a clinically relevant combination of ICPC-coded items. The total selection and arrangement procedure resulted in an FI with 36 deficits (see Additional file 1). In the baseline EMR data, the frailty software screened all patients for deficits. For some deficits, e.g., stroke, all available data for each patient were screened. For others, e.g., pneumonia, only data from the past year were considered. This strategy enables deficits to transition from ‘present’ to ‘absent’ in follow-up FI assessments, so that improvement of the FI score becomes possible over time. An ICPC-encoded deficit was present when at least one related ICPC code was registered. For single-item deficits such as ‘Heart failure’, this implied a positive ICPC-encoded item ‘K77 – Heart failure’. For multi-item deficits such as ‘Hearing impairment’, one or more of the three related ICPC-encoded items (‘H84 – Presbyacusis’, ‘H85 – Acoustic trauma’, or ‘H86 – Deafness’) were required to be positive. To calculate the polypharmacy deficit, defined as at least five different medications in chronic use, the frailty software screened for ATC codes. Three prescriptions in the past year with at least one prescription in the last six months was considered as medication in chronic use. The FI score was defined as the proportion of deficits present. For example, 12 deficits out of 36 provided a FI score of 0.33. Based on the results of the previous validation study in this primary care center, patients with an FI score of 0.08 or higher were considered as frail in the current study. In that validation study, ROC analysis demonstrated a sensitivity of 77.6 percent and a specificity of 53.5 percent for predicting adverse health outcomes (Emergency Room visits, out-of-hours GP consults, nursing home admission, and mortality) at the cut-off value of 0.08, which was considered optimal [18].

### Statistical methods

First, we calculated the descriptive statistics for baseline characteristics for the total population, for the patients grouped according to a high (≥ 4) and low (< 4) GFI score, and for the patients grouped according to a high (≥ 0.08) and low (< 0.08) FI score. Next, we constructed histograms of the distributions of the GFI and FI scores. The strength of the correlation between the FI and the GFI was calculated with Pearson’s correlation coefficient, and shared variance was calculated with R^2^. Patients were then categorised in a contingency table according to their dichotomised FI and GFI scores. Key baseline characteristics were determined for these four groups, and differences were examined between the two discrepant groups (high GFI score and low FI score; low GFI score and high FI score). Additionally, multivariate logistic regression analyses were performed to determine which baseline characteristics independently predicted this incongruence. Receiver Operator Curve (ROC) analyses were completed with the FI score as a continuous measure and the GFI score as a dichotomised variable. Finally, the mean scores for each of the four GFI sub-domains were compared between high and low FI score groups. Where appropriate, differences between groups were tested with the Pearson Chi-Square test or the Independent Samples t-test, with a p-value of < 0.05 considered significant. Analyses were performed with SPSS version 18 (SPSS, Chicago, IL).

## Results

**Figure 1:**
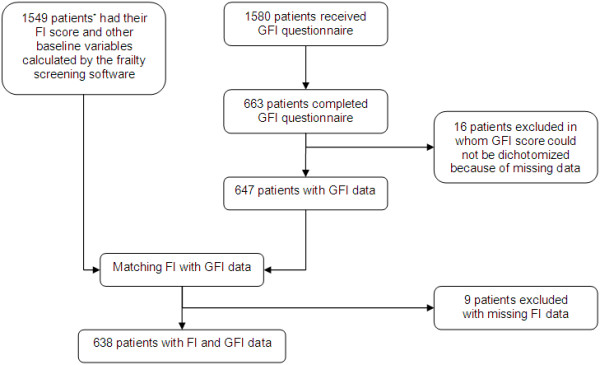
Flowchart of patient recruitment. ^* ^Of 31 patients who were born between 1 January 1951 and 30 June 1951, EMR data could not be screened by the frailty-screening software. For the pseudonymisation of personal data, birth dates were set to 1 July of the patients’ birth year. Consequently, these 31 patients were not considered as ≥ 60 years of age.

Out of 1580 eligible patients, we were able to calculate an FI score for 1549 patients (98%), and 663 patients (42%) returned the GFI questionnaire. Thus, we had 638 patients (40.4%) with complete GFI and FI data (Figure 1). Non-responders and excluded patients (N = 911) were younger than the included population (mean age non-responders and excluded patients: 71.4 years ± 9.4 SD, mean age included patients: 73.4 years ± 9.2 SD, p-value < 0.001), but they did not differ in gender, FI score, or consultation gap.

When grouped by GFI score, patients with GFI scores of four or greater were older, had higher FI scores, and shorter consultation gaps than patients with a GFI score below four (Table 1). Furthermore, patients with high GFI scores more often lived alone as a widow or following divorce, and they were less often highly educated. These trends were similar in patients grouped by FI score.

**Table 1:** Baseline characteristics for the total study population, and for high and low GFI and FI groups

	**Total**	**GFI < 4**	**GFI ≥ 4**	**p-value**	**FI < 0.08**	**FI ≥ 0.08**	**p-value**
	**(n = 638)**	**(n = 388)**	**(n = 250)**		**(n = 255)**	**(n = 383)**	
Age, mean (SD)	73.4 (9.2)	70.9 (8.2)	77.3 (9.4)	< 0.001^b^	68.7 (7.6)	76.6 (8.9)	< 0.001^b^
Females, n (%)	337 (52.8)	195 (50.3)	142 (56.8)	0.11^c^	138 (54.1)	199 (52)	0.59^c^
Frailty index score, mean (SD)	0.11 (0.08)	0.08 (0.06)	0.15 (0.08)	< 0.001^b^	0.03 (0.02)	0.15 (0.07)	< 0.001^b^
Consultation gap in days, mean (SD)	114 (347)	152 (436)	55 (81)	0.001^b^	203 (531)	55 (67.0)	< 0.001^b^
Dutch nationality, n (%)	604 (94.7)	370 (95.4)	234 (93.6)	0.33^c^	244 (95.7)	360 (94.0)	0.35^c^
Social situation							
Living alone, n (%)	56 (8.8)	28 (7.2)	28 (11.2)	0.083^c^	23 (9.0)	33 (8.6)	0.86^c^
Living with a partner, n (%)	370 (58.0)	270 (69.6)	100 (40.0)	< 0.001^c^	176 (69.0)	194 (50.7)	< 0.001^c^
Living alone as a widower or after divorce, n (%)	207 (32.4)	89 (22.9)	118 (47.2)	< 0.001^c^	54 (21.2)	153 (39.9)	< 0.001^c^
Missing, n (%)	5 (0.8)	1 (0.3)	4 (1.6)	0.061^c^	2 (0.8)	3 (0.8)	1.0^c^
Education (highest finished education)							
None or primary school, n (%)	103 (16.1)	48 (12.4)	55 (22.0)	0.001^c^	32 (12.5)	71 (18.5)	0.044^c^
Secondary school, n (%)	348 (54.5)	208 (53.6)	140 (56.0)	0.55^c^	128 (50.2)	220 (57.4)	0.072^c^
Higher education, n (%)	181 (28.4)	129 (33.2)	52 (20.8)	0.001^c^	92 (36.1)	89 (23.2)	< 0.001^c^
Missing, n (%)	5 (0.8)	3 (0.8)	3 (1.2)	0.97^c^	3 (1.2)	3 (0.8)	0.36^c^
GFI score, mean (SD)	3.2 (2.8)^a^	1.4 (1.1)	6.2 (2.0)	< 0.001^b^	1.8 (1.9)	4.2 (2.8)	< 0.001^b^
GFI score ≥ 4, n (%)	250 (39.2%)	-	-	-	39 (15.3)	211 (55.1)	< 0.001^c^

^a ^n total population = 623 for calculation of mean GFI score because 15 of the patients in whom it could be determined with certainty whether they had a GFI score ≥ 4 had missing values for some GFI questions. ^b ^Differences were evaluated with the Independent Samples t-test. ^c ^Differences were evaluated with the Pearson Chi-Square test. *FI*, Frailty index.

**Figure 2:**
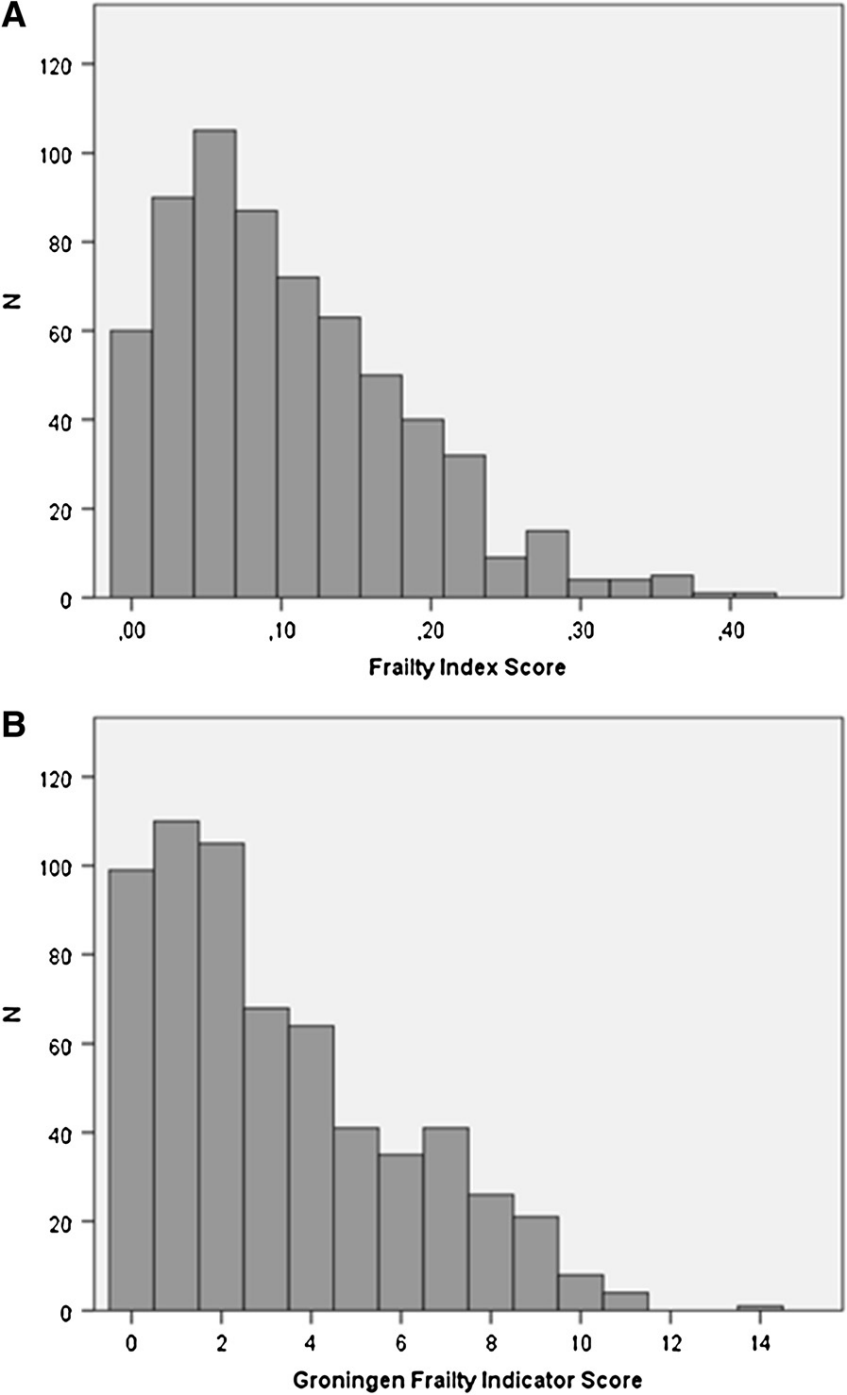
**FI and GFI score distributions. A**. FI score distribution. **B**. GFI score distribution.

Both the FI and GFI scores showed a left-skewed distribution in the study sample (Figure 2). The GFI and FI scores showed a moderate positive, linear correlation (Pearson’s correlation coefficient = 0.544, p-value < 0.001). In patients aged 60–70 years old, Pearson’s correlation coefficient was 0.522 (p < 0.001), and in patients aged 80 years and older, Pearson’s correlation coefficient was 0.431 (p = 0.001). Next, we constructed a contingency table using a cut-off value for frailty of 0.08 for the FI, and four for the GFI. With these dichotomised scores, 84.7% of patients with a low FI score also had a low GFI score. In patients with a high FI score, 55.1% also had a high GFI score (Table 2). When key baseline characteristics were compared between the two discrepant groups in the contingency table, patients in the group with a low FI score and a high GFI score were more often female, and were more often living alone as a widower or after a divorce than patients with a high FI score and a low GFI index score (Table 3). Using multivariate logistic regression, we found that in patients with a low FI score, living alone as a widower or after a divorce increased the risk of having a high GFI score. In patients with a low GFI score, older age and living alone as a widower or after a divorce increased the risk of having a high FI score (Table 4). Patients with high FI scores had higher mean scores on the physical, cognitive, social, and psychological domains of the GFI than patients with low FI scores (Table 5). The ROC analysis demonstrated that we could adequately predict that a randomly selected patient from the high-GFI-score group would also have a high FI score (AUC 0.78, 95% CI 0.74 to 0.82).

**Table 2:** Two by two contingency table of FI versus GFI

	**GFI ≥ 4**	**GFI <4**	**Total**
FI ≥ 0.08	211 (55.1%)	172 (44.9%)	383 (100%)
FI < 0.08	39 (15.3%)	216 (84.7%)	255 (100%)
Total	250	388	638

**Table 3:** Key characteristics based on FI-GFI group

	**Group 1:**	**Group 2:**	**Group 3:**	**Group 4:**	**Comparison of groups 3 and 4: p-value**
	**FI < 0.08 &**	**FI ≥ 0.08 &**	**FI ≥ 0.08 &**	**FI < 0.08 &**	
	**GFI < 4**	**GFI ≥ 4**	**GFI < 4**	**GFI ≥ 4**	
N (%)	216 (33.9)	211 (33.1)	172 (27.0)	39 (6.1)	-
Age, mean (SD)	68.0 (7.2)	78.2 (9.3)	74.6 (8.0)	72.4 (8.6)	0.12^a^
Females, n (%)	117 (52.2)	111 (53.6)	80 (48.8)	29 (67.4)	0.011^b^
Consultation gap in days, mean (SD)	222 (571)	47 (54)	65 (78)	96 (160)	0.075^a^
Living alone as a widower or after divorce, n (%)	35 (16.2)	99 (46.9)	54 (31.4)	19 (48.7)	0.040^b^
Primary education or less, n (%)	25 (11.6)	48 (22.7)	23 (13.4)	7 (17.9)	0.46^b^

^a ^Differences were evaluated with the Independent Samples t-test. ^b^ Differences were evaluated with the Pearson Chi-Square test.

**Table 4:** Independent predictive capacity of baseline characteristics for a high GFI or FI score

**Multivariate analysis I (prediction of high GFI score in patients with a low FI score)**			
	**Odds ratio**	**95% CI**	**p-value**
**Age**	1.042	0.996– 1.090	0.072
**Sex**	1.847	0.831 – 4.105	0.13
**Consultation gap in months**	0.958	0.889 – 1.033	0.27
**Living alone as a widower or after divorce**	3.797	1.760 – 8.194	0.001
**Primary education or less**	1.010	0.363 – 2.809	0.99
**Multivariate analysis II (prediction of high FI score in patients with a low GFI score)**			
	**Odds Ratio**	**95% CI**	**p-value**
**Age**	1.059	1.023 – 1.097	0.001
**Sex**	0.998	0.576 – 1.729	0.99
**Consultation gap in months**	0.923	0.848 – 1.004	0.062
**Living alone as a widower or after divorce**	2.149	1.122 – 4.114	0.021
**Primary education or less**	0.689	0.315 – 1.509	0.35

Effects are depicted per year increase in age and per month increase in consultation gap. Male gender, not living alone as a widower or after divorce, and having other education above primary education were taken as the reference values. *CI*, Confidence interval.

**Table 5:** Mean GFI domain scores per FI group

	**FI < 0.08**	**FI ≥ 0.08**	**Significance**
	**n = 255**	**n = 383**	**p-value**
Physical GFI domain			
n	250	378	
mean (SD)	0.60 (0.95)	1.90 (1.55)	< 0.001^a^
Cognitive GFI domain			
n	254	380	
mean (SD)	0.26 (0.44)	0.47 (0.50)	< 0.001^a^
Social GFI domain			
n	254	378	
mean (SD)	0.47 (0.87)	1.10 (1.18)	< 0.001^a^
Psychological GFI domain			
n	255	381	
mean (SD)	0.42 (0.72)	0.79 (0.86)	< 0.001^a^

^a ^Differences were evaluated with the Independent Samples t-test. Numbers per group differ because 15 patients have incomplete data on one or more GFI domains. Number of questions and score range per domain: Physical domain: 9 questions, score range 0–9; Cognitive domain: 1 question, score range 0–1; Social domain: 3 questions, score range 0–3, Psychological domain: 2 questions, score range 0–2.

## Discussion

### Summary

In this study, we demonstrated that, whereas both measures are extensively validated with regard to their measurement of the frailty concept, the FI based upon routine primary care data and the GFI only moderately overlap in the identification of frailty in older patients in the primary care setting [9,23,24]. Whereas most patients with few health deficits also report few problems in their daily lives, just over half of patients with multiple health deficits also report having multiple problems in their daily lives. This result illustrates that the FI and GFI cover different aspects or stages of frailty. This is supported by the results of a recent study demonstrating that ADL impairment in bathing, cooking and managing medication occurred only in about 25% of participants with a high FI score [25]. However, there may also be confounding factors that influence the correlation between the FI and the GFI, for example, the variation in self-management abilities between patients [26]. Furthermore, the GFI is a self-report instrument. Certain coping strategies or cognitive impairments might prompt the patient to report fewer problems than might actually exist, distorting the relationship between the GFI and FI. Finally, social vulnerability may influence the observed correlation between the FI and GFI, as a recent study demonstrated an increased absolute mortality risk in fit people with increased social vulnerability [27]. This is in line with the observation in our study that living alone as a widower or after a divorce is associated with high FI and GFI scores.

### Strengths and limitations

Our study has several strengths. First, we investigated two multifactorial frailty measures that are easy to implement in daily practice, both of which could serve as an initial screening tool before a comprehensive geriatric assessment [2]. Thus, our study, which was conducted with a representative sample of community-dwelling older patients, has relevant and generalisable results [28]. Second, we demonstrated that the FI and GFI are related to several baseline factors that themselves are linked to frailty, supporting the validity of both measures [5]. Third, we demonstrated that patients with high FI scores have higher mean scores on all GFI domains, not only on the physical GFI domain. Finally, 39% of our patients had a GFI score of four or higher, which is comparable to the 39-46% found in previous studies [13,26].

Our study also has some limitations. First, some selective response may have occurred among first generation immigrants due to illiteracy or a language barrier. Since these patients report more chronic conditions and a poorer self-rated health, the correlation between the FI and GFI may have been stronger in this subgroup [29]. Second, the ‘oldest old’ may experience a greater decrease in daily functioning with fewer deficits than the ‘youngest old’, resulting in a weaker correlation between the GFI and FI. This was confirmed by a lower Pearson’s correlation coefficient in patients of 80 years and older, compared to patients aged 60–70 years old. Third, our response rate was 42%. This was lower than the 77% response rate in a comparable population after one reminder [13], but comparable to the response rate of 45% in another study that did not send reminders [26]. The low response rate illustrates the practical limitations of the use of the GFI as a first step in frailty screening, but with the use of reminders, the GFI appears feasible in daily practice. Fourth, to define frailty we used a cut-off score of four for the GFI [12,26]. However, this cut-off score may also include ‘pre-frail’ patients and may be a reason to raise the minimum score for frailty [30]. Furthermore, our FI score cut-off value of 0.08 was based on a previous study in the same primary care center, in which we were the first to develop the FI measure from routine primary care data [18]. The use of routine primary care data resulted in a narrower FI score range compared to that in other studies [9]. Both an unexpectedly low prevalence of deficits identified in routine healthcare data and the fact that this study’s FI consists almost exclusively of comorbidities may have contributed to this narrow score range, and the FI and its cut off values may need to be adjusted accordingly. Finally, cognitive loss is not always identified as a deficit as a result of the corresponding ICPC codes not being registered properly. Because cognitive problems are strongly related to frailty, encoding in routine practice requires careful attention [31,32].

### Comparison with existing literature

Depending on the definition, the prevalence of frailty varies widely from 5% to 58% [33]. Some recent studies have demonstrated the continued lack of consensus in defining frailty and the limited value of currently available frailty measures for screening and diagnosis in daily practice [34,35]. However, others have concluded that the FI seems best suited for clinical use, and that an FI based on ICPC coded primary care data is associated with the risk of adverse health outcomes [18,36]. Screening and early, proactive care is essential, and with currently available frailty measures, identification of frailty does enable targeted interventions in primary care [37-39]. By exploring, for the first time, the relationship between the GFI and an FI score derived from routine healthcare data, our results contribute to the development of a frailty-screening strategy that meets the needs of primary care providers.

### Implications for research and practice

Taking the different focus of the FI and the GFI into account, we hypothesize that a two-step frailty-screening strategy could be useful to provide optimal proactive primary care for older patients. For several reasons, the FI would be the preferred first step; it uses administrative data readily available for all patients, it can be implemented as an easy-to-use software application in daily clinical practice, and it adequately predicts adverse health outcomes [9,18]. As a second step, the GFI could identify patients who also experience multiple problems in daily life besides having a high FI score. The response rate of 42% in our study is suboptimal for implementing the GFI as a frailty screening measure, and needs to be improved. However, a previous study using one reminder demonstrated a response rate of 77%. In addition, the GFI could be filled in by patients while visiting the GP, which will increase response rate as well. In patients with a high FI score and low GFI score, evaluation by the GP, reviewing medication and consultation pattern, will be sufficient. Patients with high scores on both measures might benefit from a comprehensive geriatric assessment and tailored, proactive care by a geriatric nurse. Some may question the complexity of this approach, as GFI questionnaire data may also be incorporated as deficits in the FI score. However, we think the sequential two step screening approach is the most efficient approach to personalised elderly care. Implementing GFI screening only for patients with a high FI score would result in a considerably lower work load of posting questionnaires, sending reminders, or filling in questionnaires together with patients in the primary care center, while our results show that this approach would still identify the majority of patients with a high GFI score. Second, a two-step screening process would enable the primary care practices to carefully allocate geriatric nursing care resources to those patients in highest need, as reflected by a high GFI score.

The only restriction of this approach is that patients that do not return the GFI questionnaire must be followed up because they might be care avoiders. In the U-PROFIT trial, we are currently examining the effect of this two-step screening strategy on the quality of life and daily functioning of frail older people [40].

## Conclusions

The FI and the GFI moderately overlap in identifying frailty in community-dwelling older patients. To provide optimal proactive primary care, we suggest an initial FI screening in routine healthcare data, followed by a GFI questionnaire for patients with a high FI score or otherwise at high risk as the preferred two-step frailty screening process in primary care.

## Competing interests

The authors declare that they have no competing interests.

## Authors’ contributions

ID, NB, NJW, MJS and MEN contributed to the study concept and design. ID was responsible for drafting this manuscript. ID and GK collected the data. ID, NB, GK and MJCE were involved in data analysis, interpretation and manuscript review. NJW, MJS and MEN contributed to thorough revision of the manuscript, and all authors read and approved the final version.

## Pre-publication history

The pre-publication history for this paper can be accessed here:

http://www.biomedcentral.com/1471-2296/14/64/prepub

## Supplementary Material

Additional file 1 GFI. Click here for file

Additional file 2 FI deficits. Click here for file
