# Supplementary material for: Identifying frailty: do the Frailty Index and Groningen Frailty Indicator cover different clinical perspectives? a cross-sectional study
Source: BMC Fam Pract. 2013 May 21;14:64. doi: 10.1186/1471-2296-14-64 (PMC3665587; doi:10.1186/1471-2296-14-64)
Supplement: Additional file 1 — GFI. [file 1471-2296-14-64-S1.docx]

### Additional file 1 – Groningen Frailty Indicator questionnaire

**1.** Are you able to carry out these tasks single-handedly and without any help? (The use of help

resources such as a walking stick, walking frame or wheelchair is considered to be independent.)

- Shopping

🞎 yes

🞎 no

- Walking around outside (around the house or to the neighbours)

🞎 yes

🞎 no

- Dressing and undressing

🞎 yes

🞎 no

- Going to the toilet

🞎 yes

🞎 no

**2.** What mark do you give yourself for physical fitness? (Scale 0 to 10)

Circle the number:

**0 1 2 3 4 5 6 7 8 9 10**

**3.** Do you experience problems in daily life due to poor vision?

🞎 yes, a lot of problems

🞎 yes, some problems

🞎 no, no problems

**4.** Do you experience problems in daily life due to being hard of hearing?

🞎 yes, a lot of problems

🞎 yes, some problems

🞎 no, no problems

**5.** During the last 6 months have you lost a lot of weight unwillingly?

(3 kg in 1 month or 6 kg in 2 months)

🞎 yes

🞎 no

**6.** Do you take 4 or more different types of medicine?

🞎 yes

🞎 no

**7.** Do you have any complaints about your memory?

🞎 yes

🞎 sometimes

🞎 no

**8.** Do you sometimes experience emptiness around yourself?

🞎 yes

🞎 sometimes

🞎 no

**9.** Do you sometimes miss people around yourself?

🞎 yes

🞎 sometimes

🞎 no

**10.** Do you sometimes feel abandoned?

🞎 yes

🞎 sometimes

🞎 no

**11.** Have you recently felt downhearted or sad?

🞎 yes

🞎 sometimes

🞎 no

**12.** Have you recently felt nervous or anxious?

🞎 yes

🞎 sometimes

🞎 no

**Scoring:**

Questions 1: Yes = 0; No = 1

Question 2: 0-6 = 1; 7-10 = 0

Questions 3-6: No = 0; Yes = 1

Question 7: No = 0; Sometimes = 0; Yes = 1

Questions 8-12: No = 0; Sometimes = 1; Yes = 1
