# Supplementary material for: Identifying frailty: do the Frailty Index and Groningen Frailty Indicator cover different clinical perspectives? a cross-sectional study
Source: BMC Fam Pract. 2013 May 21;14:64. doi: 10.1186/1471-2296-14-64 (PMC3665587; doi:10.1186/1471-2296-14-64)
Supplement: Additional file 2 — FI deficits. [file 1471-2296-14-64-S2.docx]

### Additional file 2 – Frailty Index deficits

| **Deficit** | **Deficit name** | **Deficit**  **Prev. (%)** | **ICPC*** | **ICPC-Label** | **Days^⯎^** | **Item**  **Prev. (%)^◆^** |
| --- | --- | --- | --- | --- | --- | --- |
| 1 | General complaints | 10.6 | A01 | Pain general/multiple sites | 365 | 2.1 |
|  |  |  | A04 | Weakness/tiredness general | 365 | 4.0 |
|  |  |  | A05 | General deterioration | 365 | 0.4 |
|  |  |  | A28 | Limited function/disability (NOS) | - | 0 |
|  |  |  | B28 | Limited function/disability (blood, blood forming) | - | 0 |
|  |  |  | B80 | Iron deficiency anaemia | 365 | 1.8 |
|  |  |  | B81 | Anaemia, Vitamin B12/folate def. | 365 | 0.9 |
|  |  |  | B82 | Anaemia other/unspecified | 365 | 0.9 |
|  |  |  | D28 | Limited function/disability (digestive) | - | 0.1 |
|  |  |  | F28 | Limited function/disability (eye) | - | 0.3 |
|  |  |  | H28 | Limited function/disability (ear) | - | 0 |
|  |  |  | K28 | Limited function/disability (circulatory) | - | 0 |
|  |  |  | L28 | Limited function/disability (musculoskeletal) | - | 0.8 |
|  |  |  | N28 | Limited function/disability (neurological) | - | 0 |
|  |  |  | P28 | Limited function/disability (psychological) | - | 0 |
|  |  |  | P78 | Neuraesthenia/surmenage | 365 | 0.3 |
|  |  |  | R28 | Limited function/disability (respiratory) | - | 0.1 |
|  |  |  | S28 | Limited function/disability (skin) | - | 0 |
|  |  |  | T28 | Limited function/disability (metabolic, endocrine, nutrition) | - | 0 |
|  |  |  | U28 | Limited function/disability (urinary) | - | 0.1 |
|  |  |  | X28 | Limited function/disability (female, genital) | - | 0 |
|  |  |  | Y28 | Limited function/disability (male, genital) | - | 0 |
|  |  |  | Z28 | Limited function/disability (social) | - | 0.1 |
| 2 | Neoplasm - other | 10.9 | A79 | Malignancy NOS |  | 0 |
|  |  |  | B72 | Hodgkin’s disease | - | 0.3 |
|  |  |  | B73 | Leukaemia | - | 0.3 |
|  |  |  | B74 | Malignant neoplasm blood other | - | 0.1 |
|  |  |  | D74 | Malignant neoplasm stomach | - | 0.1 |
|  |  |  | D76 | Malignant neoplasm pancreas | - | 0.1 |
|  |  |  | D77 | Malig. neoplasm digest other/NOS | - | 0.4 |
|  |  |  | F74 | Neoplasm of eye/adnexa | - | 0.1 |
|  |  |  | H75 | Neoplasm of ear | - | 0 |
|  |  |  | K72 | Neoplasm cardiovascular | - | 0 |
|  |  |  | L71 | Malignant neoplasm musculoskeletal | - | 0.3 |
|  |  |  | N74 | Malignant neoplasm nervous system | - | 0 |
|  |  |  | R84 | Malignant neoplasm bronchus/lung | - | 0.7 |
|  |  |  | S77 | Malignant neoplasm of skin | - | 4.6 |
|  |  |  | T71 | Malignant neoplasm thyroid | - | 0.1 |
|  |  |  | U75 | Malignant neoplasm of kidney | - | 0.3 |
|  |  |  | U76 | Malignant neoplasm of bladder | - | 0.9 |
|  |  |  | U77 | Malignant neoplasm urinary other | - | 0.1 |
|  |  |  | X75 | Malignant neoplasm cervix | - | 0.2 |
|  |  |  | X76 | Malignant neoplasm breast female | - | 2.3 |
|  |  |  | X77 | Malignant neoplasm genital other (f) | - | 0.7 |
|  |  |  | Y78 | Malignant neoplasm male genital / mammae | - | 0.2 |
| 3 | Incontinence | 11.0 | D17 | Incontinence of bowel | - | 0.9 |
|  |  |  | U04 | Incontinence urine | - | 7.3 |
|  |  |  | X87 | Uterovaginal prolapse | - | 3.6 |
| 4 | GI / Liver disease | 5.9 | D72 | Viral hepatitis | - | 0.4 |
|  |  |  | D97 | Cirrhosis / liver disease NOS | - | 0.9 |
|  |  |  | D75 | Malignant neoplasm colon/rectum | - | 1.8 |
|  |  |  | D85 | Duodenal ulcer | 365 | 1.2 |
|  |  |  | D86 | Peptic ulcer other | 365 | 0.9 |
|  |  |  | D94 | Chronic enteritis/ulcerative colitis | - | 1.0 |
| 5 | Oesophagus disease | 5.8 | D84 | Oesophagus disease | 365 | 5.8 |
| 6 | Visual impairment | 9.7 | F83 | Retinopathy | - | 1.6 |
|  |  |  | F94 | Blindness | - | 0.4 |
|  |  |  | F84 | Macular degeneration | - | 2.6 |
|  |  |  | F93 | Glaucoma | - | 5.5 |
| 7 | Cataract | 13.4 | F92 | Cataract | - | 13.4 |
| 8 | Hearing impairment | 8.8 | H84 | Presbyacusis | - | 5.8 |
|  |  |  | H85 | Acoustic trauma | - | 0.4 |
|  |  |  | H86 | Deafness | - | 2.8 |
| 9 | Respiratory problems | 5.7 | K02 | Pressure/tightness of heart | 365 | 1.2 |
|  |  |  | R02 | Shortness of breath/dyspnoea w/o K02 | 365 | 2.3 |
|  |  |  | R81 | Pneumonia | 365 | 2.4 |
| 10 | Angina pectoris | 11.2 | K74 | Angina pectoris | 365 | 11.2 |
| 11 | Myocardial disease | 6.3 | K75 | Acute myocardial infarction | 365 | 5.7 |
|  |  |  | K76 | Other / chronic ischaemic heart disease | - | 0.7 |
| 12 | Heart failure | 5.3 | K77 | Heart failure | - | 5.3 |
| 13 | Atrial fibrillation/flutter | 8.2 | K78 | Atrial fibrillation/flutter | 365 | 8.2 |
| 14 | Hypertension - uncomplicated | 35.8 | K86 | Hypertension uncomplicated | 365 | 35.8 |
| 15 | Hypertension - complicated | 8.8 | K87 | Hypertension complicated | - | 8.8 |
| 16 | Dizziness | 8.1 | A06 | Fainting/syncope | 365 | 1.6 |
|  |  |  | H82 | Vertiginous syndrome / labyrinthitis | 365 | 4.6 |
|  |  |  | K88 | Postural hypotension | 365 | 0.4 |
|  |  |  | N17 | Vertigo/dizziness | 365 | 1.7 |
| 17 | TIA / CVA | 8.9 | K89 | Transient cerebral ischaemia | 365 | 3.9 |
|  |  |  | K90 | Stroke/cerebrovascular accident | - | 5.2 |
| 18 | Vascular disease | 8.0 | K91 | Atherosclerosis | - | 1.0 |
|  |  |  | K92 | other PVD | - | 3.3 |
|  |  |  | K93 | Pulmonary embolism | 365 | 0.7 |
|  |  |  | K94 | Phlebitis/thrombophlebitis | 365 | 1.1 |
|  |  |  | K99 | Cardiovascular disease other | - | 2.7 |
| 19 | Fracture /  Osteoporosis | 11.3 | A80 | Trauma/injury NOS | 365 | 1.1 |
|  |  |  | L72 | Fracture: radius/ulna | 365 | 0.5 |
|  |  |  | L73 | Fracture: tibia/fibula | 365 | 0.5 |
|  |  |  | L74 | Fracture: hand/foot bone | 365 | 0.3 |
|  |  |  | L75 | Fracture: femur | 365 | 0.9 |
|  |  |  | L76 | Fracture: other | 365 | 1.1 |
|  |  |  | L95 | Osteoporosis | - | 8.0 |
| 20 | Arthritis /  Osteoarthrosis | 7.7 | L88 | Rheumatoid arthritis / related condition | - | 1.7 |
|  |  |  | L89 | Osteoarthrosis of hip | - | 3.6 |
|  |  |  | L91 | Osteoarthrosis other / related condition | - | 2.7 |
| 21 | Osteoarthrosis knee | 6.2 | L90 | Osteoarthrosis of knee | - | 6.2 |
| 22 | Neurologic disease | 7.1 | N86 | Multiple sclerosis | - | 0.2 |
|  |  |  | N99 | Neurological disease, other | - | 0.7 |
|  |  |  | N99 | Migraine | 365 | 0.9 |
|  |  |  | N87 | Parkinsonism, Parkinson’s disease | - | 1.3 |
|  |  |  | N88 | Epilepsy | - | 1.5 |
|  |  |  | N94 | Peripheral neuritis/neuropathy | - | 3.0 |
| 23 | Depression | 8.0 | P03 | Feeling depressed | 365 | 2.0 |
|  |  |  | P76 | Depressive disorder | 365 | 6.1 |
| 24 | Sleep disturbance | 11.5 | P06 | Sleep disturbance | 365 | 11.5 |
| 25 | Cognitive impairment | 5.6 | P20 | Memory / concentration / orientation disturbance | 365 | 2.3 |
|  |  |  | P85 | Mental retardation | - | 0.1 |
|  |  |  | P70 | Dementia / Alzheimer’s disease | - | 3.3 |
| 26 | Psychiatric problems / Substance abuse | 5.1 | P71 | Organic psychosis other | 365 | 0.5 |
|  |  |  | P72 | Schizophrenia | - | 0.1 |
|  |  |  | P73 | Affective psychosis | 365 | 0.4 |
|  |  |  | P74 | Anxiety disorder/anxiety state | 365 | 1.5 |
|  |  |  | P15 | Chronic alcohol abuse | - | 1.6 |
|  |  |  | P16 | Acute alcohol abuse | 365 | 0.1 |
|  |  |  | P17 | Tobacco abuse | - | 1.1 |
|  |  |  | P18 | Medication abuse | 365 | 0 |
|  |  |  | P19 | Drug abuse | 365 | 0 |
| 27 | COPD | 8.8 | R91 | Chronic bronchitis / bronchiectasis | - | 0.7 |
|  |  |  | R95 | Chronic obstructive pulmonary disease | - | 8.1 |
| 28 | Asthma | 5.8 | R96 | Asthma | - | 5.8 |
| 29 | Skin problems | 6.8 | S70 | Herpes zoster | 365 | 1.5 |
|  |  |  | S91 | Psoriasis | - | 1.7 |
|  |  |  | S97 | Chronic ulcer skin | 365 | 3.7 |
| 30 | Weight problems | 4.9 | T05 | Feeding problem of adult | 365 | 0.1 |
|  |  |  | T07 | Weight gain | 365 | 0.1 |
|  |  |  | T08 | Weight loss | 365 | 1.3 |
|  |  |  | T83 | Overweight | - | 0.8 |
|  |  |  | T82 | Obesity | - | 2.7 |
| 31 | Thyroid disorders | 6.2 | T85 | Hyperthyroidism/thyrotoxicosis | 365 | 1.2 |
|  |  |  | T86 | Hypothyroidism/myxoedema | 365 | 5.0 |
| 32 | Diabetes mellitus | 18.8 | T90 | Diabetes mellitus | - | 18.8 |
| 33 | Urinary disease | 7.5 | U99 | Urinary disease, other | - | 7.5 |
| 34 | Prostate problems | 5.4 | Y77 | Malignant neoplasm prostate | - | 2.1 |
|  |  |  | Y85 | Benign prostatic hypertrophy | - | 3.3 |
| 35 | Social problems | 5.7 | Z01 | Poverty/financial problem | 365 | 0.1 |
|  |  |  | Z03 | Housing/neighbourhood problem | 365 | 0.4 |
|  |  |  | Z04 | Social cultural problem | 365 | 0.2 |
|  |  |  | Z29 | Social problem NOS | 365 | 0.4 |
|  |  |  | Z12 | Relationship problem with partner | 365 | 0.5 |
|  |  |  | Z14 | Partner illness problem | 365 | 1.0 |
|  |  |  | Z15 | Loss/death of partner problem | - | 3.4 |
| 36 | Polypharmacy | 28.8 | - | - | 365 | 28.8 |

* Dutch ICPC-1 version as currently used in general practices

^⯎^ ‘365 days’ indicates that the belonging item is only considered present when registered at least once in the past year. For items without the ‘365 days’ indication, all time presence is considered.

^◆^ The reported item prevalences on which the frailty index deficit arrangement has been based come from this study´s primary care centre, but are based on EMR data of November 2008.

Prev. = Prevalence
